# Apigenin Combined With Gefitinib Blocks Autophagy Flux and Induces Apoptotic Cell Death Through Inhibition of HIF-1α, c-Myc, p-EGFR, and Glucose Metabolism in EGFR L858R+T790M-Mutated H1975 Cells

**DOI:** 10.3389/fphar.2019.00260

**Published:** 2019-03-22

**Authors:** ZiSheng Chen, Dongbo Tian, Xiaowen Liao, Yifei Zhang, Jinghua Xiao, Weiping Chen, Qingxia Liu, Yun Chen, Dongmin Li, Lianyu Zhu, Shaoxi Cai

**Affiliations:** ^1^ Department of Respiratory and Critical Care Medicine, Chronic Airways Diseases Laboratory, Nanfang Hospital, Southern Medical University, Guangzhou, China; ^2^ Department of Respiratory Medicine, The Sixth Affiliated Hospital of Guangzhou Medical University, Qingyuan People’s Hospital, Qingyuan, China; ^3^ Department of Neurology, Jiangmen Hospital of Traditional Chinese Medicine Affiliated to Jinan University, Jiangmen, China

**Keywords:** AMPK, autophagy, apoptosis, combination, apigenin, gefitinib, mutation, non-small cell lung cancer

## Abstract

Cancer cells are characterized by abnormally increased glucose uptake and active bio-energy and biosynthesis to support the proliferation, metastasis, and drug resistant survival. We examined the therapeutic value of the combination of apigenin (a natural small-molecule inhibitor of Glut1 belonging to the flavonoid family) and gefitinib on epidermal growth factor receptor (EGFR)-resistant mutant non-small cell lung cancer, to notably damage glucose utilization and thus suppress cell growth and malignant behavior. Here, we demonstrate that apigenin combined with gefitinib inhibits multiple oncogenic drivers such as c-Myc, HIF-1α, and EGFR, reduces Gluts and MCT1 protein expression, and inactivates the 5′ adenosine monophosphate-activated protein kinase (AMPK) signaling, which regulates glucose uptake and maintains energy metabolism, leading to impaired energy utilization in EGFR L858R-T790M-mutated H1975 lung cancer cells. H1975 cells exhibit dysregulated metabolism and apoptotic cell death following treatment with apigenin + gefitinib. Therefore, the combined apigenin + gefitinib treatment presents an attractive strategy as alternative treatment for the acquired resistance to EGFR-TKIs in NSCLC.

## Introduction

Due to the high number of tobacco consumers in China, i.e., over 300 million male smokers and more than 740 million people exposed to second-hand smoke (Yang et al., 2015). By 2015, the number of newly diagnosed cases of lung cancer had reached 0.73 million (Chen et al., 2015). Non-small cell lung cancer (NSCLC), comprising 80–85% of lung cancer, is the major histological subtype of this cancer (Meza et al., 2015).

The mutation rate of epidermal growth factor receptor (EGFR) in mainland China population has reached 50.2%, and the activating mutation rate is 48.0% (Shi et al., 2015). In all EGFR mutations of NSCLC patients, deletion of EGFR exon 19 and mutation of EGFR L858R exon 21 account for 85–90%. NSCLC patients have high response rates to EGFR tyrosine kinase inhibitors (EGFR-TKIs) (Rotow and Bivona, 2017). However, acquired resistance to these EGFR-TKIs frequently develops after 9–13 months exposure, and mutation of EGFR T790M accounts for 60% of this acquired resistance associated with progressive disease after first response to TKIs (Janne et al., 2015). Thus, a new effective strategy is urgently needed.

Cancer cells have high rates of glucose metabolism, which is regulated primarily by c-Myc, HIF-1α (Phan et al., 2015), and the glucose transporter 1 (Glut1) (Adekola et al., 2012). The reason for this is that c-Myc and HIF-1α directly activate the transcription of glycolytic enzymes (Denko, 2008; Phan et al., 2015). Cross-talk between oncogenic pathways and glucose metabolism may provide opportunities for therapeutic strategies against TKI resistance in NSCLC. Studies have shown that Glut1 over-expression is associated with drug resistance (Shimanishi et al., 2013) and poor overall survival (Tan et al., 2017). EGFR-TKIs such as gefitinib inhibit glucose uptake *in vitro* (Makinoshima et al., 2014) and models of EGFR mutant NSCLC *in vivo* (Su et al., 2006). However, earlier studies found that TKIs such as erlotinib do not sufficiently reduce the nuclear HIF-1α and c-Myc protein levels in PC-9 (EGFR exon 19 deletion) xenograft mouse model when used alone, but a combination of erlotinib + cisplatin produced significant nuclear HIF-1α and c-Myc downregulation and tumor size inhibition (Lee and Wu, 2015). This demonstrates the importance and efficacy of combination treatment in cancer. So far, the regulation of HIF-1α and c-Myc in glucose metabolism in the context of TKI resistance in NSCLC has not been well researched, and hence, the regulatory mechanisms involved remain obscure.

The prevailing evidence indicates that flavonoids, which are present in many grains, fruits, and vegetables, may reduce the risk of cancer through its antioxidant effects and by eliminating free radicals derived from DNA damage and inflammation (Sung et al., 2016). Apigenin, a 4′,5,7-trihydroxyflavone compound, is a natural flavone mainly derived from Apium genus such as Chinese celery and parsley (Sung et al., 2016). Previous studies have demonstrated that apigenin reduces both mRNA and protein expression of Glut1 in a concentration and time-dependent pattern (Melstrom et al., 2008); hence, it is involved in the control of glucose uptake (Park, 1999). At present, the anti-tumor mechanism of apigenin has been shown to involve the induction of autophagy, apoptosis, immune response, inhibition of cell cycle, migration, and invasion of cancer cells (Yan et al., 2017). Studies have shown that apigenin reduces nuclear c-Myc and intracellular HIF-1α protein level in a dose-dependent manner, which leads to significant tumor inhibition (Liu et al., 2005; Shukla et al., 2007). Moreover, the combination of apigenin + paclitaxel presents a synergistic effect that increases cancer cell apoptosis (Xu et al., 2011). Whether targeting both c-Myc and HIF-1α to regulate glucose utilization changes the dynamics of the apoptotic mechanism in EGFR mutant intrinsic TKIs resistance in NSCLC is unknown. Here, we hypothesized that a combination of apigenin + gefitinib might provide a superior pharmacological effect for killing the NSCLC cells with intrinsic TKI resistance.

In this study, we emphasized the necessity and effectiveness of combined use in resistant cancer treatment and, for the first time, revealed that apigenin + gefitinib combination inhibits AMPK signaling pathway and oncogenic drivers c-Myc, HIF-1α, and EGFR and damages the glucose uptake and utilization on EGFR mutant-resistant NSCLC cells. Apigenin + gefitinib is a very clinically promising combination use.

## Materials and Methods

### Cell Culture and Reagents

Human EGFR-TKIs resistant NSCLC cell line NCI-H1975 (#No. CRL-5908TM) was purchased from ATCC (American type culture collection; Manassas, VA, USA). Immortalized human epithelial cell line BEAS-2B was also obtained from ATCC. Human lung squamous cell carcinoma and immortalized human liver cell line 95-D and HL7702, respectively, were purchased from Shanghai cell bank affiliated to the Chinese Academy of Sciences (Shanghai, China). H1975 and HL7702 cells were maintained in RPMI-1640 medium (Sigma, St. Louis, MO, USA) containing 10% fetal bovine serum (FBS, Gibco, USA). BEAS-2B and 95-D cells were cultured in Dulbecco’s modified Eagle’s medium (DMEM, Sigma, St. Louis, MO, USA) supplemented with 5 and 10% fetal bovine serum, respectively, in a humidified atmosphere containing 5% CO_2_ at 37°C.

Osimertinib (AZD-9291), 10058-F4 (Myc-Max disruptor), and STF-31 (a specific Glut-1 inhibitor) were purchased from MedChem Express (Monmouth Junction, NJ, USA). KC7F2, gefitinib, and cisplatin were obtained from APExBIO (Houston, TX, USA). Chloroquine (CQ) was acquired from Sigma (St. Louis, MO, USA). Rapamycin was obtained from Selleck Chemicals (Houston, TX, USA). Cell Counting Kit-8 (CCK-8) was purchased from Beyotime Biotech (Shanghai, China).

### Cell Proliferation and Migration and Colony Formation Assays

The anti-proliferative effect of gefitinib, apigenin (Solarbio, Beijing, China), and the combination of the two compounds was determined by CCK-8 assay. H1975, 95-D, BEAS-2B, and HL7702 were treated with gefitinib, apigenin, and combination at the indicated concentrations and times. Apigenin and gefitinib were reconstituted in dimethyl sulfoxide (DMSO) to 100 and 10 mM stock, respectively, and stored at −20°C in the dark. Absorbance was detected at 450 nm by a Microplate Reader (SpectraMax 190, Molecular Devices, USA).

H1975 cells were grown to reach ~70–80% confluence as a monolayer in six-well plates. A single-layer scratch was created with one abacterial 200 microliter pipette tip across the well center. The well was gently washed twice with a cold medium to remove the adherent cells. Immediately, images from each well were recorded, and the recording was repeated at 72 h to measure the wound process. Cell migration distance to the scratched area was determined by Image J software.

Single H1975 cells were obtained, and totally 250 cells were seeded in each well of six-well plates overnight. The cells were treated with gefitinib, apigenin, and combination at the indicated concentration and time. The media were removed, and then cells were rinsed with 10 ml phosphate buffered saline (PBS) before fixation for 15 min in 75% ethanol. A 0.5% crystal violet solution was added followed by incubation at room temperature (RT) for 2 h to visualize.

### Flow Cytometry Analysis of the Cell Cycle and Apoptosis

The cell cycle kit and Annexin V-FITC/propidium iodide (PI) apoptosis kit (4A Biotech, Beijing, China) were used according to the manufacturer’s instructions. H1975 cells were harvested and fixed in 1 ml 75% ethanol for 24 h. The supernatant was discarded, and the cells were re-suspended in 0.5 ml cold PBS containing 10 μg/ml RNase and 20 μg/ml PI stock solution. Subsequently, they were transferred to FACS tubes and incubated in the dark for 30 min at RT. Cell cycle was assayed by flow cytometry (BD, Biosciences) at 488 nm. For apoptosis assay, adherent cells were harvested after 24 h of treatment and re-suspended in 100 μl binding buffer (1 × 10^6^ cells/ml), then incubated with 5 μl Annexin V–FITC for 5 min in the dark at RT. A 10 μl PI (20 μg/ml) and 400 μl binding buffer were added to the cells, which were then immediately analyzed by flow cytometry at 630 and 525 nm.

### Lactate Production and ATP Production Assays

For lactate production assay, H1975 cells were plated in 96-well plates at the density of 1 × 10^4^ cells/well and incubated overnight. Then, they were treated with gefitinib, apigenin, and combination at the indicated concentrations for 24 h. All procedures were in accordance with the manufacturer’s protocol for L-Lactate Assay Kit I (Eton Bioscience, San Diego, CA, USA). Absorbance was measured at 490 nm using a SpectraMax 190 microplate reader. For ATP production assay, H1975 cells were seeded in 12-well plates at a density of 8 × 10^4^ cells/well and incubated overnight. Following the manufacturer’s protocol for ATP Assay Kit (Beyotime Biotech, Shanghai, China), absorbance was measured using a Luminescence Microplate Reader (Infinite M200PRO, TECAN). A standard curve was prepared with the given L-Lactate and ATP concentrations and was used to determine the actual concentration of L-Lactate and ATP in the samples.

### Determination of Glucose Consumption and Mitochondrial Mass

Glucose consumption and mitochondrial mass in H1975 cells were determined as previously described in the study of Phan et al. (2015). Briefly, H1975 cells were cultured in glucose-free DMEM supplemented with 10% fetal bovine serum and containing 100 μg/ml of green fluorescent glucose analog 2-(*N*-(7-nitrobenz-2-oxa-1,3-diazol-4-yl) amino)-2-deoxyglucose (2-NBDG, Molecular Probes, Cayman) for a duration spanning from 0 to 30 min. 2-NBDG consumption was analyzed using a flow cytometer, and the data were analyzed by a FlowJo X software. Mitochondrial mass was determined by staining with a green fluorescent dye MitoTracker Green FM (MTG, Molecular Probes, Invitrogen) and then analyzed using a fluorescent microscope (ZEISS, Germany).

### Western Blot Analysis

H1975 cells were lysed by a strong RIPA buffer (Beyotime, Shanghai, China) containing PMSF and phosphatase inhibitor (Beyotime, Shanghai, China). Ultrasonic cracking for 10–15 s was performed to complete the lysis of the cells and to break DNA. BCA Protein Assay Kit (Beyotime, Shanghai, China) was used to determine the amount of protein in the supernatant. The samples were boiled with 5× SDS loading buffer for 5 min. Then equal amounts of protein were subjected to SDS-PAGE followed by transfer of the proteins to a PVDF membrane using the wet transfer method. The membranes were blocked with 5% non-fat milk for 1 h at RT. The primary antibodies used in this study included anti-Cdc2 (phospho Thr161), anti-CDK4, anti-Cyclin D1 (Immunoway), anti-E-cadherin, anti-MMP9, anti-Bcl-2 (Bioss), anti-MMP2, anti-BIM, anti-caspase-3, anti-PARP-1, anti-Glut3, anti-MCT1, anti-HIF-1α (Santa Cruz), anti-Bax, anti-GAPDH, anti-Glut1, anti-Glut4, anti-LDHA, anti-PDK1, anti-p-EGFR, anti-c-Myc, anti-p-AMPKα, anti-β-Tubulin (CST), anti-p62, anti-Kras (Proteintech), anti-LC3B (Novus Biologicals), and anti-β-actin (Ray Antibody Biotech). The membranes were incubated with these antibodies at 4°C overnight. Thereafter, they were rinsed in TBST three times each round for 5 min at RT. The membranes were then incubated with diluted horseradish peroxidase (HRP) labeled secondary antibody (CST and Proteintech, respectively) at the recommended dilution (1/2,000 and 1/4,000, respectively) in TBST. Finally, the blots were quantified by ECL Plus detection using a chemiluminescence system (Bio-RAD, USA).

## Results

### A Combination of Apigenin With Gefitinib Inhibited EGFR L858R-T790M Mutant H1975 Cells

**Figure 1:**
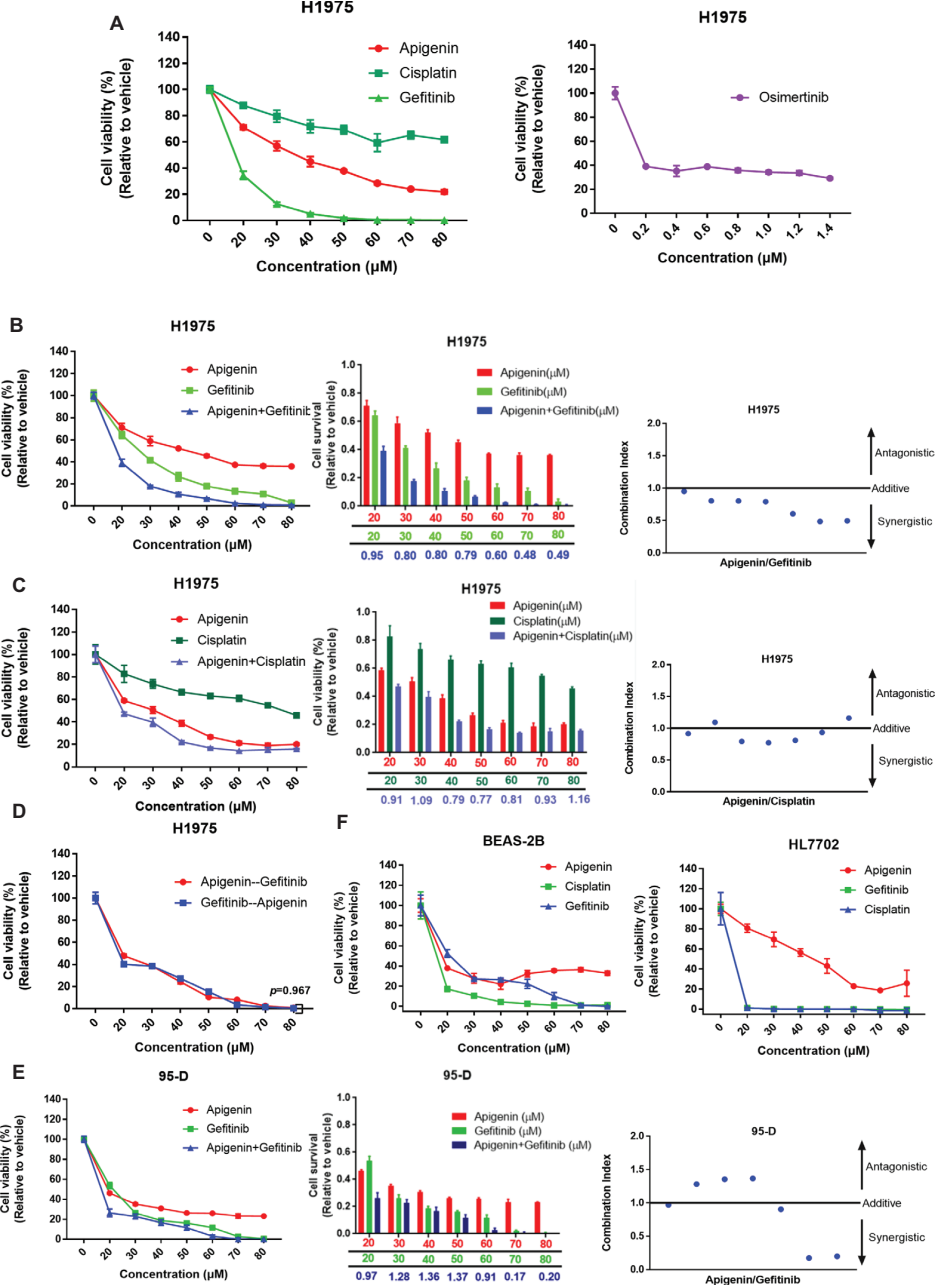
Apigenin and gefitinib combination reduces the growth of EGFR mutant resistance H1975 cells. **(A)** H1975 cells were cultured with apigenin, gefitinib, and cisplatin at the indicated concentrations for 72 h, and osimertinib was used as a positive control. **(B)** H1975 cells were incubated with apigenin, gefitinib, and combination at the indicated concentrations for 48 h and combination index for H1975 cells incubated with apigenin + gefitinib. **(C)** H1975 cells were treated with apigenin, cisplatin, and combination at the indicated concentrations for 72 h and combination index for H1975 cells incubated with apigenin + cisplatin. **(D)** H1975 cells were incubated with apigenin and gefitinib at the indicated concentrations for 24 h, and then gefitinib and apigenin were added at the indicated concentrations for 48 h, respectively. **(E)** 95-D cells were treated with apigenin, gefitinib, and combination at the indicated concentrations for 72 h and combination index for 95-D cells treated with apigenin + gefitinib. **(F)** BEAS-2B and HL7702 cell lines were incubated with apigenin, gefitinib, and cisplatin at the indicated concentrations for 72 h. Cell viability was measured using CCK-8 kit, and absorbance was measured by a Microplate Reader at 450 nm. Combination index (CI) was calculated according to the Chou-Talalay method (Chou and Talalay, 1984), and CI values < 1 represent a synergistic drug-drug interaction. Statistical analysis (*p < 0.05) was performed using the GraphPad Prism 7.0 and unpaired Student’s *t* test. All data are presented as the mean ± SD. Error bars indicate ± SD.

EGFR-TKI insensitive cell line NCI-H1975 contains mutations of L858R, ∆E746-A750, and T790M at exon 20 (Wang et al., 2016b). These cells are highly insensitive to EGFR-TKIs. For example, for gefitinib and erlotinib, IC50 is 12 μM; however, sensitive cell lines such as PC9 have an extremely low IC50 of 4 nM (Sharma et al., 2007). Here, we initially tested the survival of cells after treatment with different concentrations of gefitinib, apigenin, and cisplatin for 72 h, using osimertinib as the positive control. Significant cytotoxic activity was observed in H1975 cells in all compounds except cisplatin (Figure 1A). Then, we further determined whether apigenin would synergize with gefitinib to decrease the cell viability. We tested increasing doses of apigenin and gefitinib alone or in combination with the cells (Figure 1B) and also tested increasing doses of apigenin and cisplatin alone or in combination with H1975 cells (Figure 1C). Results of the combined index showed that apigenin synergized with gefitinib in the H1975 cells (Figure 1B). However, this synergy was not observed when apigenin was combined with cisplatin, since even high concentrations of these combinations could not completely kill the cells (Figure 1C). Next, we tested the effect of different application sequences of apigenin and gefitinib on the proliferation of H1975 cells. Analysis of the cell viability tests showed that sequential dosing of apigenin and gefitinib did not have significant effects on proliferation (Figure 1D). We similarly used the human high metastatic lung squamous cancer cell line 95-D, which is an EGFR mutant wild type, to determine the effect of increasing doses of apigenin and gefitinib alone or in combination. Analysis of the results of the combination index revealed that apigenin did not synergize with gefitinib in 95-D cells (Figure 1E). In addition, analysis of cell toxicity of apigenin, gefitinib, and cisplatin showed that apigenin toxicity was much less than that of gefitinib and cisplatin in BEAS-2B and HL7702 cell lines (Figure 1F). These findings show that apigenin combined with gefitinib selectively induces a beneficial response in EGFR L858R-T790M mutant H1975 cells.

### A Combination of Apigenin and Gefitinib Induces the G_0/_G_1_ Cell Cycle Arrest and Cell Metastasis in H1975 Cells

**Figure 2:**
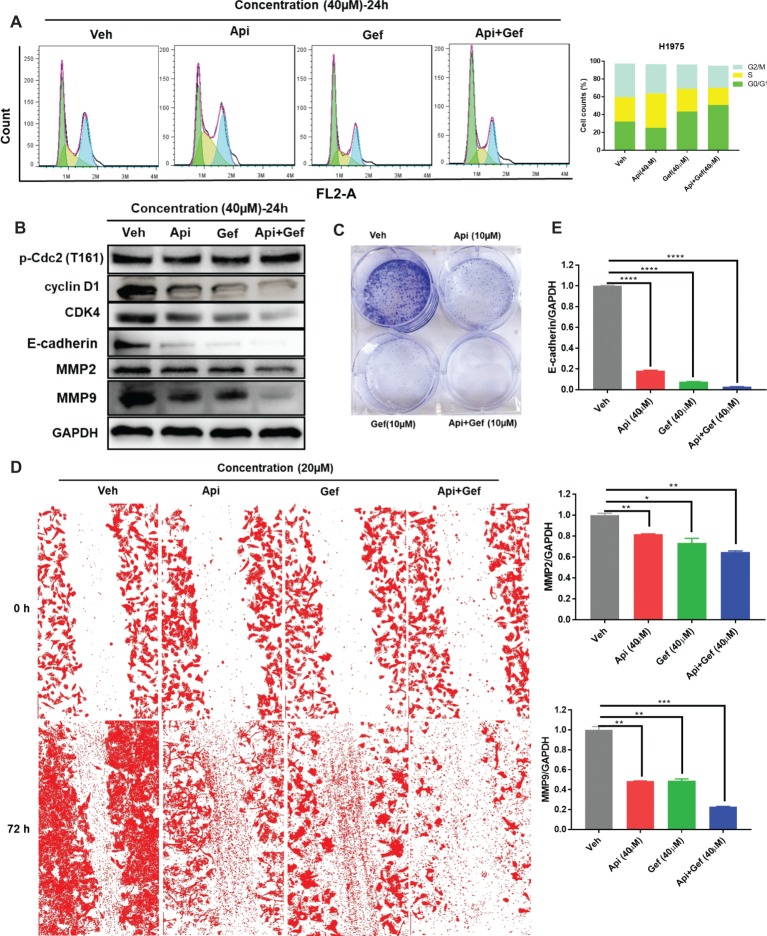
Treatment with apigenin and gefitinib combination causes the cell cycle arrest in the G_0_/G_1_ phase and inhibits proliferation and metastasis of H1975 cells. **(A)** H1975 cells were incubated with apigenin, gefitinib, and combination at the indicated concentrations for 24 h, followed by PI staining and flow cytometry analysis for the cell cycle progression. **(B)** Cells treated with apigenin, gefitinib, and combination at the indicated concentrations for 24 h were lysed and used for western blot analysis with antibodies against cell cycle-related proteins and metastasis-associated proteins, including Cyclin D1, CDK4, p-Cdc2 (T161), E-cadherin, MMP2, and MMP9. **(C)** The blots clearly showed that treatment with apigenin, gefitinib, and the combination for 8 days inhibited cell colony formation, but the compound combination induced a significant anti-clonogenic effect than others. **(D)** Representative phase contrast images from H1975 live cell recordings of each condition are shown at 0 and 72 h. Images were acquired with an inverted phase-contrast microscope at 100× magnification for photomicrograph. Cell scratch assay images were analyzed by ImageJ software. **(E)** Apigenin + gefitinib treatment significantly downregulated E-cadherin, MMP2, and MMP9 expression at 24 h. Statistical significance (*p < 0.05; **p < 0.01; ***p < 0.001, ****p < 0.0001) was analyzed using GraphPad Prism 7.0 with unpaired Student’s *t* test. All data are presented as the mean ± SD. Error bars indicate ± SD. (Veh: vehicle; Api: apigenin; Gef: gefitinib; Api + Gef: apigenin + gefitinib).

We then postulated that a combination of apigenin and gefitinib may have an additive effect and induce alterations to the cell cycle progression, cell proliferation, and migration in H1975 cells. To test these, we determined cell cycle arrest after treatment with apigenin, gefitinib, and a combination of the two at the indicated concentration for 24 h. Analysis of the cell cycle was performed by using flow cytometry. As shown (Figure 2A), treatment with a combination of the compounds induced H1975 cell cycle arrest in G_0_/G_1_ phase. To further explore the molecular mechanisms involved in the cell cycle arrest, we performed western blot analysis. We found that the G_0_/G_1_ phase arrest of the cell cycle following treatment of cells with compound combinations was accompanied with significant downregulation of Cyclin D1 and CDK4. p-Cdc2 (T161) is one of the protein marker associated with G_2_/M phase (Morgan, 1995). In this study, western blot analysis showed no difference in p-Cdc2 (T161) expression (Figure 2B). A colony formation assay was used to investigate cell proliferation, and the results showed that a combination of the compounds remarkably inhibited colony formation (Figure 2C). To understand the role of combined treatment on NSCLC metastasis, we carried out a scratch wound assay. Migration of H1975 cells into the wound area was measured by acquiring images at time intervals ranging from 0 to 72 h. The results showed that apigenin, gefitinib, and the combination inhibited cell migration, but the combination induced much higher cell migration inhibition than individual compounds leading to cell death (Figure 2D). We therefore investigated the molecular mechanisms responsible for this observation. Western blot analysis indicated that all these compounds induced a decrease in E-cadherin, MMP2, and MMP9 expression, and the decrease was the highest in the compound combination group (Figures 2B, E). Collectively, these results demonstrate that the combined use of apigenin + gefitinib induces H1975 cell cycle arrest in G_0_/G_1_ phase and reduces cell proliferation and metastasis.

### Apigenin Combined With Gefitinib Induces Apoptosis in H1975 Cells

**Figure 3:**
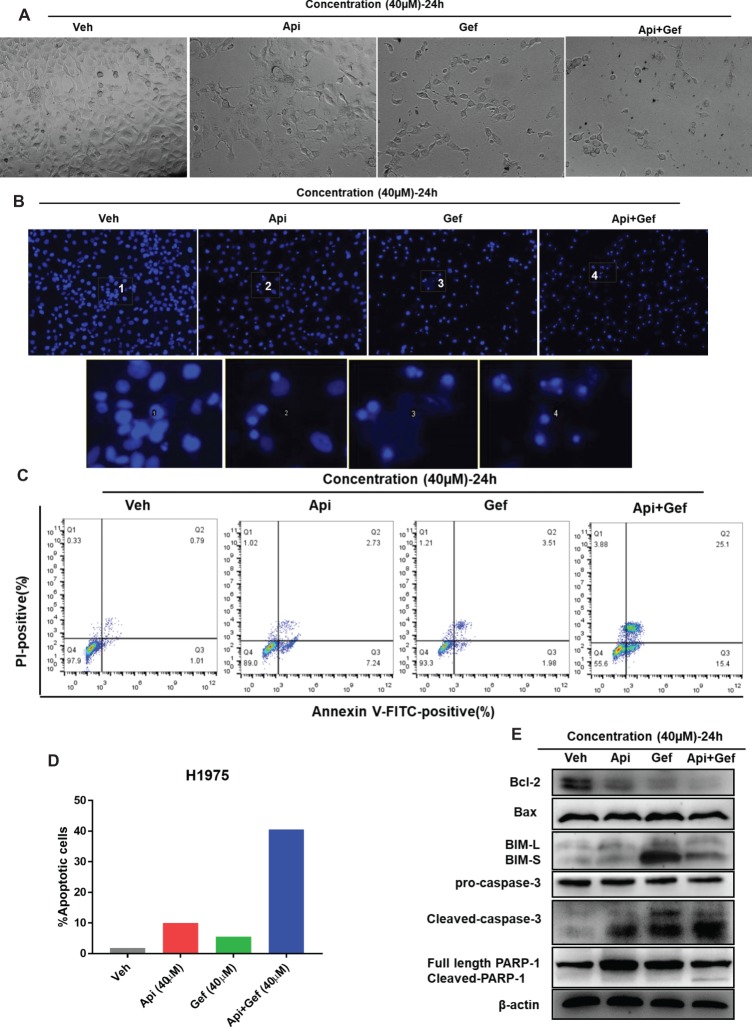
Apigenin combined with gefitinib augments apoptotic cell death in H1975 cells. **(A)** The morphology of the H1975 cells is observed in the vehicle and in each treatment group under the inverted phase contrast microscope after 24 h. **(B)** H1975 cells apoptotic bodies were determined with each treatment condition at 24 h by fluorescence microscopy after Hoechst33342 staining (100×). **(C)** H1975 cells were subjected to Annexin-V-FITC/PI dual staining following compound treatment for 24 h. Apoptosis was determined by flow cytometry, and apoptosis rate was measured as shown in **(D)**. **(E)** The protein expressions of Bcl-2, Bax, BIM, caspase-3, and PARP-1 were analyzed using western blot for 24 h. (Veh: vehicle; Api: apigenin; Gef: gefitinib; Api + Gef: apigenin + gefitinib).

To examine the level of apoptosis as a process of decreasing the number of cells in proliferation and migration assay, we first observed the changes in each configuration of the treated H1975 cells by inverted phase contrast microscopy. As shown (Figures 3A, B), the majority of H1975 cells were spindle shaped and had pseudopodia. Apigenin treatment resulted in cell morphology changes that were oval shaped and pseudopod elongation. Similarly, gefitinib changed the cell morphology to oval shape and cell detachment, while Hoechst33342 staining showed high nuclear DNA condensation. Apigenin combined with gefitinib showed characteristic apoptotic morphology such as cell shrinkage, round shape, nuclear DNA condensation, marginalization or fragmentation, and detachment. To further investigate the apoptosis rate, we performed flow cytometry analysis using Annexin V-FITC/PI dual staining. The proportion of apoptotic H1975 cells markedly increased after 24 h of combined treatment (Figures 3C, D). Caspase-cleaved PARP is a key effector of apoptosis. The western blotting analysis demonstrated that the combination of compounds significantly increased cleaved-caspase-3 and cleaved-PARP-1 expression after 24 h treatment. In addition, the anti-apoptotic protein Bcl-2 was significantly downregulated, the pro-apoptotic protein BIM was upregulated, and Bax was remarkably increased after gefitinib treatment (Figure 3E). Collectively, these results indicate that apigenin combined with gefitinib significantly induced H1975 cell apoptosis.

### Apigenin Combined With Gefitinib Compromises Metabolism in H1975 Cells

**Figure 4:**
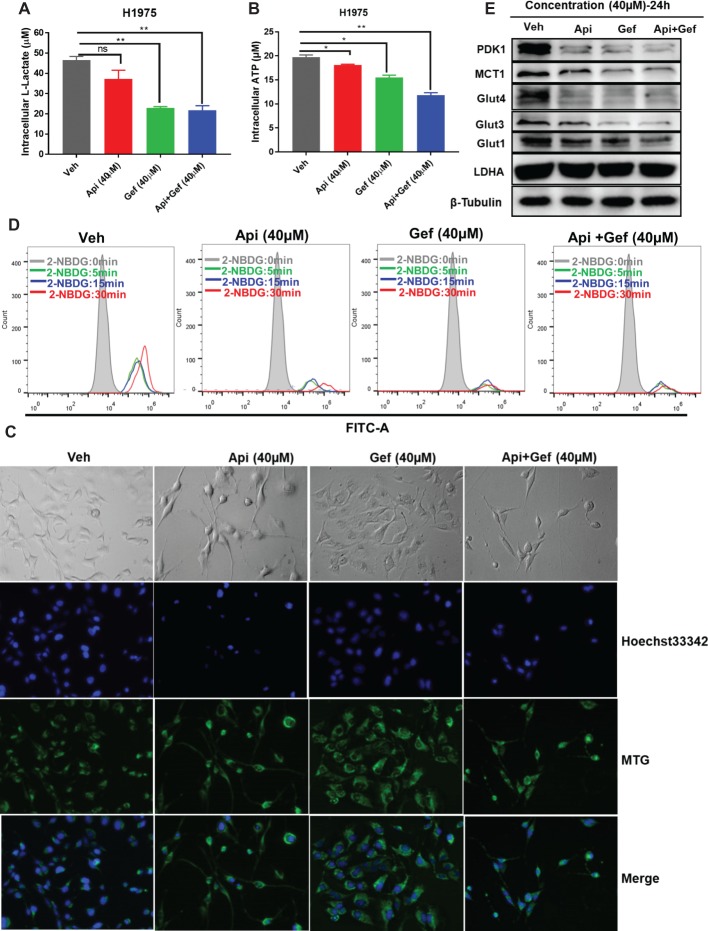
Reduced glucose uptake and lactate export caused by compound treatment in H1975 cells. Metabolite analysis of the H1975 cells treated with different compounds. Changes in metabolites levels were plotted for intracellular **(A)** lactate and for **(B)** ATP production. **(C)** H1975 cells were treated with different conditions for 60 h, and inverted fluorescence microscope was used to acquire fluorescent images after staining with Hoechst33342 and MTG. **(D)** H1975 cells were treated with different conditions for 48 h, and glucose uptake was measured after incubation with 2-NBDG at the indicated time points. 2-NBDG fluorescence intensity was measured using a flow cytometer. **(E)** Cell lysates were used to test the expression of glucose and lactate metabolism-associated proteins with specific antibodies. Statistical significance (*p < 0.05; **p < 0.01) was analyzed using GraphPad Prism 7.0 with unpaired Student’s *t* test. All data are presented as the mean ± SD. Error bars indicate ± SD. (Veh: vehicle; Api: apigenin; Gef: gefitinib; Api + Gef: apigenin + gefitinib).

We further investigated the effect of the compounds on the metabolism of H1975 cells. *In vitro* analysis of H1975 cells showed that gefitinib and the compound combination reduced intracellular lactate and ATP production at 24 h (Figures 4A, B). We then monitored the changes in mitochondrial mass in H1975 cells after receiving single and combination treatment. MitoTracker Green is an independent mitochondrial membrane potential fluorescence dye that binds to the mitochondrion. Using the inverted fluorescence microscope, we found that the number of stained mitochondria reduced upon apigenin and combination treatment at 60 h. Simultaneously, the configuration of the cells changed to a pseudopod elongation and mitochondrial perinuclear aggregation compared with the vehicle (Figure 4C). We further used 2-NBDG to measure glucose uptake. Flow cytometry analysis of 2-NBDG at the indicated time points showed a significant decrease in glucose uptake after gefitinib and combination treatment for 48 h (Figure 4D). Additionally, we assayed the indicators of glucose uptake, lactate production, and export using western blotting analysis. We found that gefitinib and the combination markedly downregulated the expression of glucose uptake-associated proteins such as Glut1, Glut3, Glut4, PDK1, and lactate export-associated protein MCT1, but the treatments did not alter lactate production kinase LDHA expression (Figure 4E). Taken together, these findings show that a combination of apigenin and gefitinib notably impairs metabolism in H1975 cells.

### Apigenin Combined With Gefitinib Inhibits AMPK Pathway and Autophagy Flux, Leading to Augmented H1975 Apoptotic Cell Death

**Figure 5:**
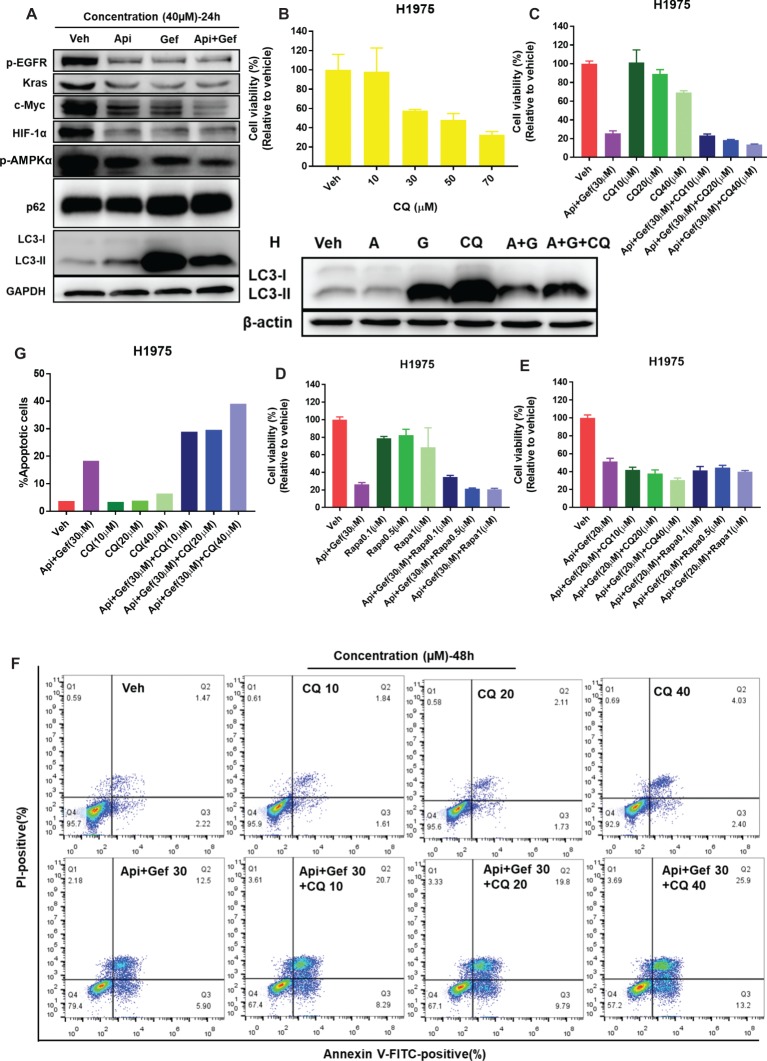
Treatment with apigenin and gefitinib combination inhibits AMPK signaling and autophagy flux, causing H1975 cell death. **(A)** H1975 cell lysates were used to test autophagy and the expression of oncogenic biomarkers such as p-EGFR, Kras, c-Myc, and HIF-1α with specific antibodies. **(B)** H1975 cells were incubated with vehicle or increasing concentrations of CQ. **(C–E)** Cell viability of H1975 cells treated with vehicle and with the indicated concentrations of apigenin + gefitinib, increasing concentrations of CQ or Rapamycin and apigenin + gefitinib + CQ or Rapamycin, respectively. For each group, cell viability was determined 48 h after the addition of compounds. Cell viability was measured using CCK-8 kit. All data are mean values ± SD of three independent experiments. **(F,G)** H1975 cell apoptosis was tested using flow cytometry, treated as shown in **(C)** for 48 h. **(H)** Lysates from H1975 cells treated with vehicle, apigenin 40 μM (A), gefitinib 40 μM (G), CQ 50 μM and apigenin + gefitinib (40 μM) + CQ 50 μM (A + G + CQ) for 36 h were probed with anti-LC3. (Veh: vehicle; Api: apigenin; Gef: gefitinib; Api + Gef: apigenin + gefitinib).

Biomarker analysis of H1975 cells indicated a strong reduction of oncogenic drivers EGFR and Kras signaling and its downstream metabolic proteins such as Glut1, Glut3, and Glut4, PDK1, and MCT1 (Figures 4E, 5A). As the transcriptional regulators of MCT1 and Glut1 (Pires et al., 2014; Sabnis et al., 2017), we found that all treatments inhibited c-Myc and HIF-1α protein expressions in H1975 cells, especially in the combination treatment (Figure 5A). The data also suggested that combined therapy induced energetic stress in H1975 cells (Figure 4B), which indicated that the AMPK signaling and autophagy were probably activated to support H1975 cell survival. We found that p-AMPKα levels in H1975 cells were decreased when treated with the compounds, especially in the combination treatment (Figure 5A). Furthermore, autophagy flux was blocked after treatment of H1975 cells with gefitinib and the combination as illustrated by the band alteration from LC3-I to LC3-II, suggesting increased expression of lipidated LC3 and p62 (Figure 5A). When treated with the combination of compounds, H1975 cells underwent apoptosis as shown by the increased cleaved-PARP-1 and cleaved-caspase-3 (Figure 3E). Examination of CQ treatment in H1975 cells demonstrated that cell growth was inhibited in a concentration-dependent manner at 48 h (Figure 5B). Furthermore, we analyzed CQ for apigenin + gefitinib combination at 48 h and found that cytotoxicity was enhanced at low concentration of the combination, but it was not observed in Rapamycin combination group (Figures 5C–E). In addition, flow cytometric analysis showed that CQ with apigenin + gefitinib combination at 48 h enhanced apoptosis in a concentration-dependent pattern (Figures 5F, G). Lastly, H1975 cells underwent autophagy flux as CQ increased LC3-II protein levels at 36 h, which were reversed by apigenin + gefitinib and apigenin + gefitinib + CQ (Figure 5H). Taken together, these results indicate that treatment with apigenin + gefitinib combination inhibits the AMPK pathway and autophagy flux, leading to enhanced H1975 apoptotic cell death.

### Regulation of p-EGFR, c-Myc, HIF-1α, and Glucose Metabolism Form the Mechanism by Which Apigenin Combined With Gefitinib Increases H1975 Cell Death

**Figure 6:**
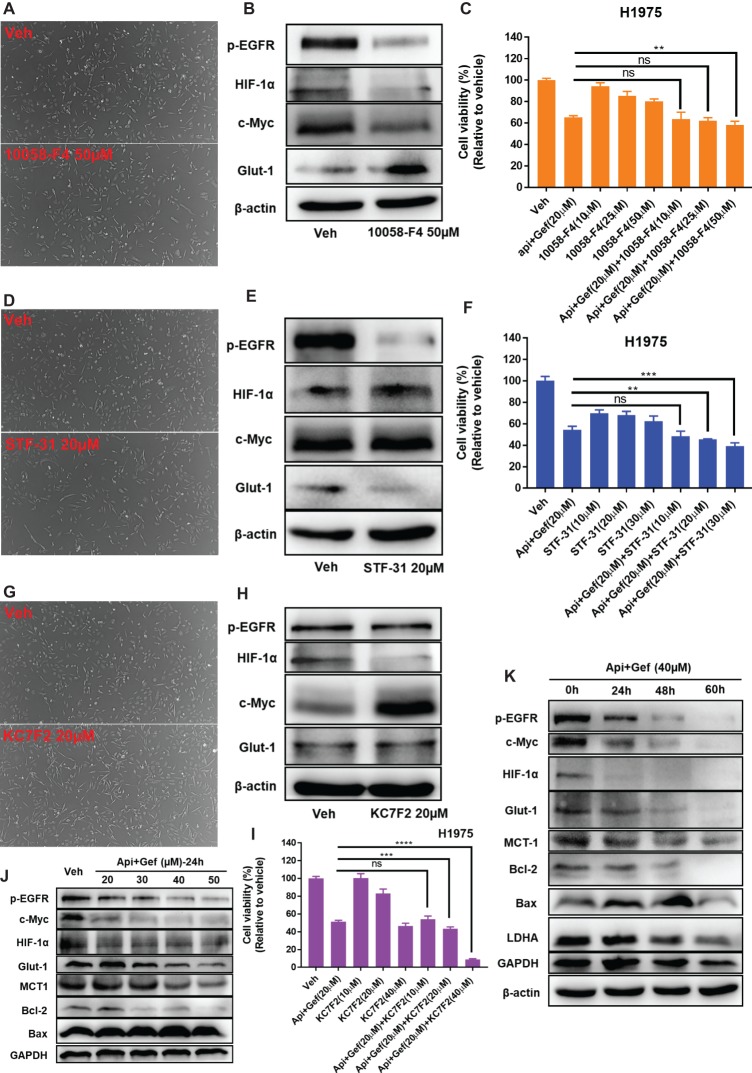
Targeting of multiple oncogenes and glycolysis increases H1975 apoptotic cell death. **(A)** The morphology of the H1975 cells is shown for the vehicle and following compound treatment at the indicated concentrations of 10058-F4, STF-31 **(D),** and KC7F2 **(G)** for 24 h under inverted phase contrast microscope. **(B,E,H,J,K)** H1975 cells were treated with different conditions, and cell lysates were used to test protein expression using specific antibodies. **(C,F,I)** Cell viability recordings for the indicated protocols for 36 h as quantified using CCK-8 assay. Statistical significance (ns: not significant; **p < 0.01; ***p < 0.001; ****p < 0.0001) was analyzed using GraphPad Prism 7.0 with unpaired Student’s *t* test. All data are presented as the mean ± SD. Error bars indicate ± SD.

Based on its ability to inhibit glucose metabolism and oncogenes, we reasoned that a combination of apigenin + gefitinib may serve as an effective strategy for EGFR-TKI resistance. Therefore, c-Myc expression was decreased by using 10058-F4 treatment and that specifically prevents the c-Myc-Max interaction and inhibits transactivation of c-Myc target gene expression. We found that 1005-F4 changed the morphology of H1975 to a more spindle-shaped and decreased p-EGFR, HIF-1α, and c-Myc expression but upregulated Glut1 protein expression (Figures 6A, B). Cell viability was significantly decreased when apigenin + gefitinib was combined with a high concentration of 10058-F4 (Figure 6C). Next, we blocked Glut1 using STF-31, a specific inhibitor of Glut1. The results showed that STF-31 treatment changed the cell morphology and markedly downregulated p-EGFR and Glut1 but did not change c-Myc and HIF-1α expression (Figures 6D, E). Cell viability following treatment with apigenin + gefitinib combined with STF-31 was decreased in a STF-31concentration-dependent manner (Figure 6F). Lastly, we blocked HIF-1α using KC7F2, a potent HIF-1 pathway inhibitor. The results revealed that KC7F2 led to a spindle-shaped morphology accompanied with a significant inhibition of HIF-1α expression and an increase in c-Myc expression. However, KC7F2 failed to downregulate p-EGFR and Glut1 expression (Figures 6G, H). Cell viability following treatment with apigenin + gefitinib combined with KC7F2 was decreased in a KC7F2 concentration-dependent manner (Figure 6I). Interestingly, western blotting analysis demonstrated that apigenin + gefitinib combination inhibited p-EGFR, HIF-1α, c-Myc, Glut1, and MCT1 in a concentration and time-dependent manner and simultaneously induced cell apoptosis through inhibition of Bcl-2 and increase in Bax expression (Figures 6J, K). These data sets indicate that selective inhibition of p-EGFR, HIF-1α, c-Myc, and glycolysis may not rescue EGFR-TKI resistance.

### Glut1, c-Myc, EGFR, and HIF-1α High Expression Are Associated With Lung Adenocarcinoma a Poor Prognosis

**Figure 7:**
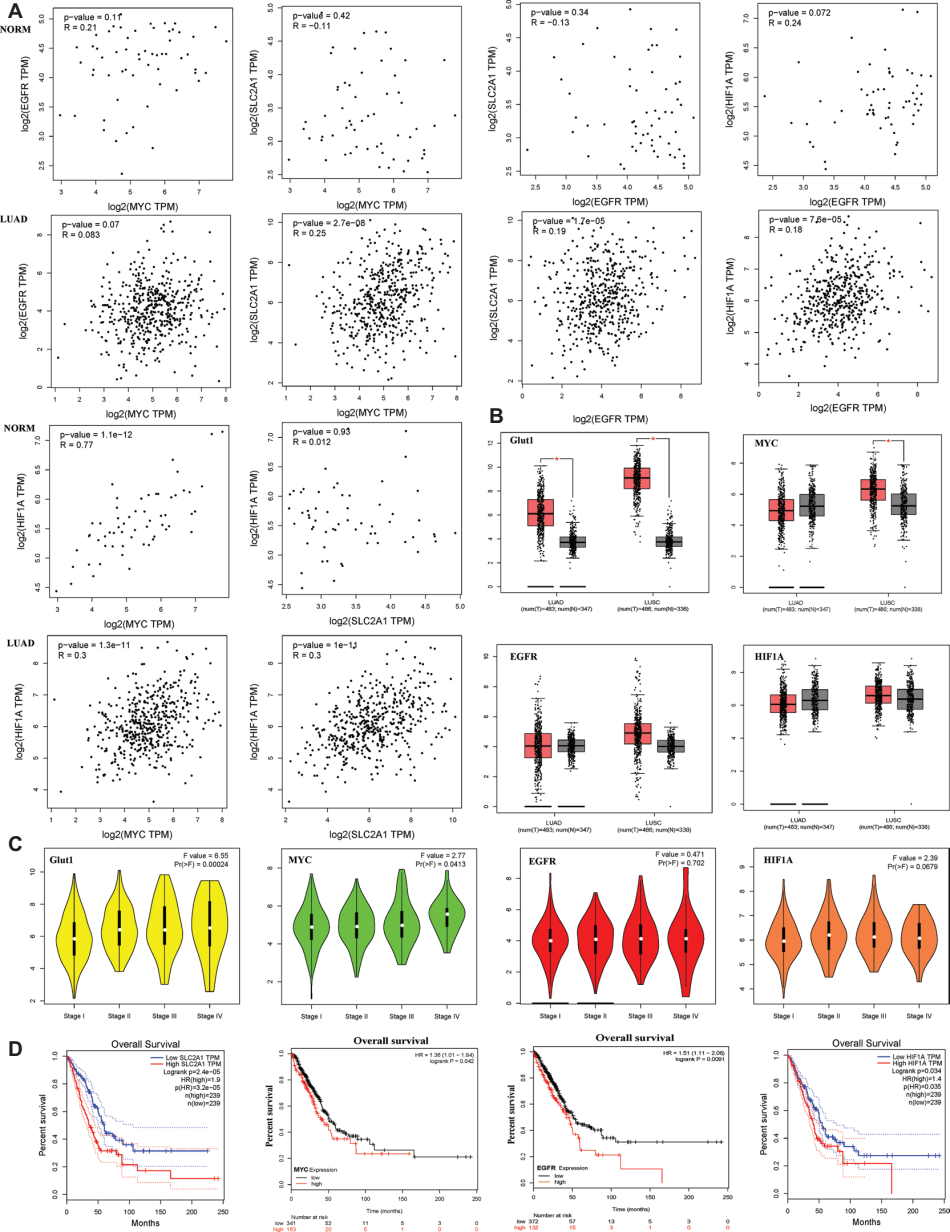
Glut1, c-Myc, EGFR, and HIF-1α high expression are associated with lung adenocarcinoma a poor prognosis. **(A)** The correlation between c-Myc, HIF-1α, EGFR, and Glut1 expression based on TCGA data in GEPIA (Tang et al., 2017) and LUAD tumor tissues as analyzed by Pearson’s correlation coefficient. **(B)** HIF-1α, c-Myc, EGFR, and Glut1 expression patterns and transcripts per million (TPM) of HIF-1α, c-Myc, EGFR, and Glut1 RNA in 483 LUAD tissues (T), 347 normal tissues (N), 486 LUSC tissues (T), and 347 normal tissues (N), respectively. Data based on GEPIA, *p < 0.05. **(C)** Glut1 and c-Myc exhibited high expression level in advanced clinical stages of LUAD (stages II–V) (*F* value = 6.55, *p* = 0.00024 and *F* value = 2.77, *p* = 0.0413). HIF-1α and EGFR showed no difference in expression level in advanced clinical stages of LUAD (stages II–V) (*F* value = 2.39, *p* = 0.0679 and *F* value = 0.471, *p* = 0.702). Data based on GEPIA, *p < 0.05. **(D)** Survival analysis of four hub genes in LUAD (HIF-1α and Glut1 based on TCGA data in GEPIA, c-Myc and EGFR was obtained from http://kmplot.com/analysis/. Statistical significance was quantified by the log-rank test). Red line symbolized the samples with highly expressed genes, and blue or black line represented the lowly expressed genes. HR: hazard ratio.

Bioinformatics analysis showed that c-Myc was correlated with HIF-1α in normal lung tissues (NORM), but it was not correlated with EGFR and Glut1. Besides, the expression of HIF-1α, EGFR, and Glut1 was not related to each other in the NORM (Figure 7A). However, in lung adenocarcinoma (LUAD), c-Myc expression was correlated with HIF-1α and Glut1, and the expression of HIF-1α, EGFR, and Glut1 was related to each other in LUAD (Figure 7A). Moreover, Glut1 expression was significantly upregulated in LUAD and lung squamous carcinoma (LUSC) compared to normal tissues, but c-Myc was upregulated in LUSC but not in LUAD. However, HIF-1α and EGFR expression were similar in LUAD and LUSC compared with their normal tissues (Figure 7B). These results indicated that Glut1 may act as a tumor suppressor in NSCLC. Further analysis of the relationship between the clinical stage and gene expression of the above proteins showed that c-Myc, HIF-1α, and Glut1 are highly expressed in the advanced clinical stages of LUAD (stages II–IV), but there was no significant relationship between EGFR expression and the stage of LUAD (Figure 7C). Finally, Kaplan-Meier curves of overall survival showed that high expression of HIF-1α, Glut1, c-Myc, and EGFR was significantly correlated with shorter overall survival in LUAD (Figure 7D).

## Discussion

**Figure 8:**
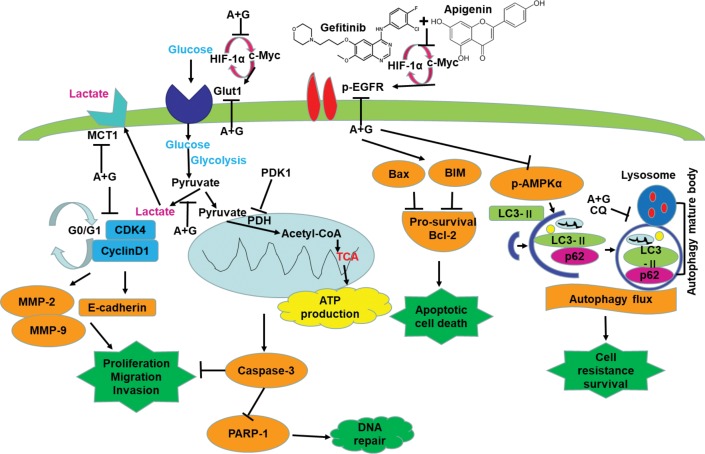
Apigenin + gefitinib combination decreases intracellular signaling and suppresses cell survival in TKI resistant H1975 cells. (A: apigenin; G: gefitinib).

In the current study, as shown in Figure 8, we show that a combination of apigenin + gefitinib in EGFR-mutated, TKI-resistant NSCLC will induce metabolic stress and energetic disturbance in H1975 cells, which lead to apoptotic cell death. We show that the combination effectively decreased expression of oncogenic drivers HIF-1α, c-Myc, and their downstream targets p-EGFR and Glut1, both of which are involved in intracellular signaling (Figures 5A, 6B, E, H). Importantly, combining the two compounds decreased HIF-1α, c-Myc, p-EGFR, and Glut1 expression in a concentration and time-dependent manner (Figures 6J, K), which was accompanied with decreased Bcl-2 and increased Bax expression (Figures 6J, K).

Potential explanations for the ability of the compound combination to increase cell death include modulation of cell cycle progression, apoptosis, and autophagy. To determine these possibilities, we first tested the cell cycle progression and found that the compound combination induced cell cycle arrest at the G_0_/G_1_ phase through inhibition of Cyclin D1 and CDK4 expression (Figures 2A, B) thereby reducing cell proliferation. In the previous study, we showed that malignancy inhibition was caused by G_0_/G_1_ cell cycle arrest (Wang et al., 2016a). We also found that combining the compounds markedly downregulated MMP-2, MMP-9, and E-cadherin expression (Figure 2B), which correlated with reduced cancer cell migration and invasion (Figures 2C, D). Clinical data indicate that high BIM expression decreases the risk of mortality and progression in EGFR-mutant NSCLC patient (Karachaliou et al., 2015). In addition, suppression of BIM expression causes NSCLC TKI resistance (Tetsu et al., 2016), whereas PARP-1 inhibitor presents a therapeutic strategy for TKI resistance in NSCLC (Li et al., 2016). Next, we investigated the mechanism of apoptosis in each treatment group of H1975 cells and found that combination therapy induced apoptosis by activating caspase-3, inactivating PARP-1, increasing BIM, and reducing Bcl-2 expression (Figure 3E).

Autophagy is a survival pathway that involves the recycling of cellular components for cellular repair and homeostasis (Mizushima and Komatsu, 2011). We found that the compound combination caused significant energetic stress by reducing intracellular ATP production (Figure 4B), glucose uptake (Figure 4D), and Gluts protein expression (Figure 4E). The failure of Glut1 inhibitor apigenin alone to suppress the growth of H1975 cells (Figures 1B, 2C) may be due to disappointedly decreased glucose uptake (Figure 4D), c-Myc, and HIF-1α expression (Figure 6E). These findings highlight the vital role of combination treatment that targets oncogenic drivers c-Myc, HIF-1α, and their downstream glycolysis-associated proteins such as Glut1, EGFR, and MCT1. These results were supported by the bioinformatics analysis, which showed that Glut1 was correlated with HIF-1α, c-Myc, and EGFR expression in LUAD (Figure 7A), although another specific Glut1 inhibitor WZB117 did not show any effect on tumor growth in LUAD A549 and H1299 xenografts of mouse model (Goodwin et al., 2017). Moreover, bioinformatics analysis showed that c-Myc expression was lower in LUAD than the normal tissues (Figure 7B), and inhibition of c-Myc resulted in increased Glut1 expression in H1975 cells (Figure 6B). The Myc/Max interaction inhibitor 10058-F4 (30 mg/kg) did not have a significant inhibitory effect on the human prostate cancer xenograft model *in vivo* (Guo et al., 2009). The HIF-1α expression is associated with TKI resistance (Minakata et al., 2012). Here, inhibition of HIF-1α resulted in increased c-Myc but no effect on p-EGFR and Glut1 expression in H1975 cells (Figure 6H). Tirapazamine is a hypoxia-activated cytotoxic prodrug, and nitroglycerin is known to block hypoxia. Yet, both drugs failed to show a survival advantage in advanced NSCLC (stages IIIB–IV) when combined with chemotherapy in a randomized phase III trials (Salem et al., 2018).

We further determined that the severe energetic stress effect of combination treatment caused the inactivation of the AMPK pathway and blocking autophagy flux. In addition, autophagy was essential to the elementary growth and survival of H1975 cells (Figure 5B), and H1975 cells experienced autophagy flux as CQ induced LC3-II expression (Figure 5H). Apoptotic cell death caused by combination treatment was enhanced by CQ in H1975 cells (Figures 5F,G). Previous studies have also shown that a combination of CQ and gefitinib, which enhances the inhibition of autophagy, is more effective in reducing the TKI resistance tumor growth (Tang et al., 2015). Based on a large number of preclinical evidence, there are many clinical trials recently present the safety and efficiency of hydroxychloroquine (HCQ), a less toxic drug than CQ, combined with TKIs or anti-angiogenesis or chemotherapy in NSCLC (NCT00977470, NCT00809237, NCT00933803, and NCT01649947).

## Conclusion

Our findings present a basis for the clinical assessment of combined targeting of oncogene-driven glycometabolism (such as inhibition of c-Myc, HIF-1α, and EGFR) and Glut1 in patients with resistance TKIs of NSCLC. Combinatorial compound led to severe energetic crisis accompanied by AMPK inactivation and the blockage of autophagy flux that resulted in metabolic stress and thereby apoptotic cell death in H1975. These types of tactic may enable the recognition of effective drug combinations and novel beneficial targets and also the project of dissimilar types of clinical trials to learn the killing of oncogene-driven cancers by drug-induced energetic-related signaling pathways.

## Author Contributions

ZC carried out all the *in vitro* studies and wrote the manuscript. DT, YZ, JX, XL, WC, QL, YC, DL, and LZ contributed to reagents, materials, and analysis tools. SC superintended the study and supplied ideas for the amendment of the manuscript.

### Conflict of Interest Statement

The authors declare that the research was conducted in the absence of any commercial or financial relationships that could be construed as a potential conflict of interest.
